# Supplementary material for: Diffusive kinks turn kirigami into machines
Source: Nat Commun. 2024 Feb 10;15:1255. doi: 10.1038/s41467-024-45602-7 (PMC10858914; doi:10.1038/s41467-024-45602-7)
Supplement: Supplementary file 1 — Supplementary Information [file 41467_2024_45602_MOESM1_ESM.pdf]

Supplementary information:

Diffusive kinks turn kirigami into machines

Shahram Janbaz and Corentin Coulais†
Institute of Physics, Universiteit van Amsterdam, 1098 XH Amsterdam, The Netherlands
†Corresponding author, C.J.M.Coulais@uva.nl

A. Geometrical design of kirigami plates

In our study, we use a thick kirigami that allows multimaterial texturing through its thickness in order to achieve strain rate dependency. The simple geometry of our kirigami design—a plate perforated with a regular pattern of parallel cut-lines—can be represented using one of its unit cells (Figs. 2a, S1a). We split the geometry of the representative unit cell into four main compartments for multimaterial texturing (Figs. 2b, S1b). In general, upon stretch, such a unit cell randomly buckles into two distinct modes: symmetric and anti-symmetric modes (Fig. 2a). Patterning with multimaterial allows us then to overcome this randomness and to control the direction of buckling by exploiting imperfections that arise from the behavior of the chosen viscoelastic polymers [1–3]. We computationally examined and analyzed the geometrical features of the representative unit cell used in our study, to ensure the manufacturability of strain rate sensitive kirigami by considering the limits of the commercially available photopolymers [4] (see section B). We were, therefore, able to manufacture viscoelastic kirigami plates that transform their geometry in different fashions in response to predicted ranges of strain rates using a polyjet 3D printer (Connex 500, Stratasys) with two soft viscoelastic photopolymers (Tango$^+$ and Agilus, Stratasys). Given the fact that these two polymers exhibit different levels of shrinkage after photo-polymerization [5], the final 3D printed material has a wavy surface with maximum curvatures close to the symmetry lines of its unit cells. In the absence of extra photo-curable soft polymers, the resulting geometrical imperfections are useful to prevent snapping in half unit cells with top Agilus material once the paired half unit cell with top Tango$^+$ exhibits viscoelastic snapping. To minimize the size of imperfection, depending on the scale of kirigami unit cells, we use a finishing thin layer of Agilus coating on the multi-texture kirigami. Finally, to further maximize the strain rate dependency in kirigami designs, we replace the area between the symmetry lines of kirigami unit cells with rigid stiff material (Vero, Stratasys) disks. The rigid areas can be also useful for carrying extra mechanisms we use in (Figs. 1, 5).

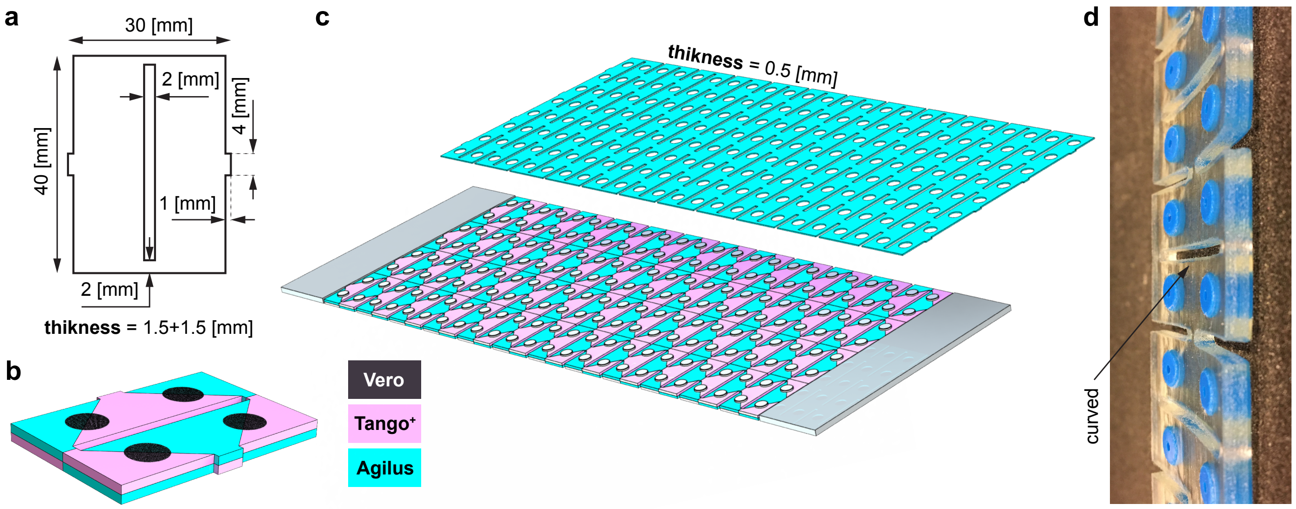

FIG. S1: **Viscoelastic kirigami plates.** (a) The schematic of the standard unit cell used in our study. (b) We split the geometry of the kirigami unit cell into four equally thick compartments for anisotropic multimaterial texturing—using two soft photopolymers Tango$^+$ and Agilus. In some designs we replace the boundary of Tango$^+$ and Agilus with Vero, which is a stiff photopolymer, to enhance the strain rate dependency and facilitate carrying objects. (c) In the specimens made of standard-size unit cells, we use a thin layer of Agilus coating to minimize the size of geometrical imperfections. (d) The curved surface of a 3D printed kirigami strip at its bottom side.

B. Strain rate dependent kirigami plates

In order to predict the manufacturability of viscoelastic kirigami using our conventional additive manufacturing technique (i.e., polyjet printing), we first used non-linear computational mechanics (Abaqus ver. 2020, Standard solver). We analyzed the post-buckling of the units cells we aim to use in our study (Figs. S2a,b). The commercial materials that we used for the fabrication of kirigami materials are different viscoelastic elastomers (i.e., Agilus and Tango$^+$, Stratasys). We, therefore, used a visco-hyperelastic material model (using the first term of Prony series) to define the visco-hyperelasticity of Agilus and TangoPlus . The material constants $C_{10A}^{\infty} = 0.080$ [MPa] and $C_{10T}^{\infty} = 0.092$ [MPa], as well as the dimensionless coefficients of Prony series $g_{1A} = 0.826$ and $g_{1T} = 0.705$, and time scales $\tau_{1A} = 0.25$ and $\tau_{1T} = 0.218$ have been chosen according to the fitting of the visco-hyperelastic material model to the stress relaxation test results of Agilus and Tango$^+$ (Figs. S2d,e). The material parameters were determined by minimizing the difference between the stress values predicted by the material model and the experimental data, assuming that the two viscoelastic polymers are incompressible [6]. Moreover, to discretize the geometry of the kirigami, we used three-dimensional elements C3D8H. A mesh convergence study has been performed to ensure the insensitivity of our computational analysis to the mesh size. The clamped-clamped condition has been realized by fixing the nodal points of one end of the kirigami while the nodal points of the moving end have the freedom to move along the axial direction of the plates. Periodic boundary condition has been satisfied by constraining the motion of the nodal points at the two free boundaries of the unit cells with respect to a reference point placed on one node. While Agilus and TangoPlus are capable of steering the buckling of kirigami, their viscoelastic properties are not sufficient for conducting snap-back in pre-stretched unit cells. Geometrical imperfections are, then, useful to enrich kirigami with such behavior. We used a half model of kirigami strip, partitioned it, and defined specific loading conditions to mimic the geometrical imperfection that appears by polyjet printing of kirigami in a nonlinear static simulation (Figs. S2f). We then introduced such geometrical imperfection to our nonlinear viscoelastic simulation to study the buckling and propagation of kink in our kirigami. To ensure a constant speed, we fixed the boundary load to the value of the load at the boundaries after 25 seconds. Our computational analysis confirms the manufacturability of our multi-texture kirigami using polyjet printing.

C. Strain rate sensitivity

Using our custom-made test bench (Fig. S3a) we show that our two kirigami designs (TATA and TAAT) exhibit robust shape-transformations at the lower and upper speed limits of our test bench (i.e., $v_{min} = 1\ mm/s$ and $v_{max} = 1000\ mm/s$). From low to fast speeds, the TAAT design exhibits two symmetric modes, and the TATA design exhibits switching from a symmetric to an anti-symmetric geometry (Figs. 2, S3, and Videos 1) which are in accordance with our computational predictions. This is expected since we have considered the existing geometrical imperfections and the effect of the cover layer on the dissimilar response of the Agilus and Tango$^+$ materials printed at the top and bottom sides of the kirigami unit cells.

At the intermediate range of speeds, we observe that both symmetric and anti-symmetric modes coexist (Fig. S3b). This coexistence is mainly due to geometrical imperfections and, less importantly, friction and boundary effects. While the TATA design exhibits an irregular mixture of symmetric and anti-symmetric modes, the TAAT design displays a longitudinally aligned bi-domain (Fig. S3b). The existence of the bi-domain is mainly due to symmetry and the collective longitudinal behavior of the unit cells. To analyze the sensitivity of buckling to loading speed, using a Matlab code, we evaluated the average values of the lateral strains by detecting and tracking the dark particles placed symmetrically over the geometry of the unit cells. Independent from the effect of material properties, both designs exhibit an identical pattern of buckling (i.e., symmetric buckling) with similar value of lateral strains ($\varepsilon = -30\%$) when they are stretched at the low speed. At intermediate speeds, $1 < log(\frac{v}{v_{min}}) < 2$, the buckling of both designs is sensitive to loading speed. The sensitivity can be manifested by a reduction in the absolute value of lateral strains. It can be seen that the TATA design exhibits sensitive buckling over a wider range of speeds (Fig. S3c) that is mainly due to its anti-symmetric multimaterial pattern. Eventually, the high-speed mode of buckling appears at higher speeds. While the average value of the lateral strain growth to a positive value ($\varepsilon = 10\%$) in symmetric designs, the maximum value of strain is equal to the average of the lower and higher speeds strains ($\varepsilon = -10\%$). It is noteworthy to note that the analysis of lateral strains is influenced by the fact that the traced particles are placed on top of the kirigami plates. This can be a valuable point while a kirigami plate serves as a flexible substrate for carrying 3D objects as the source of mechanical functionalities.

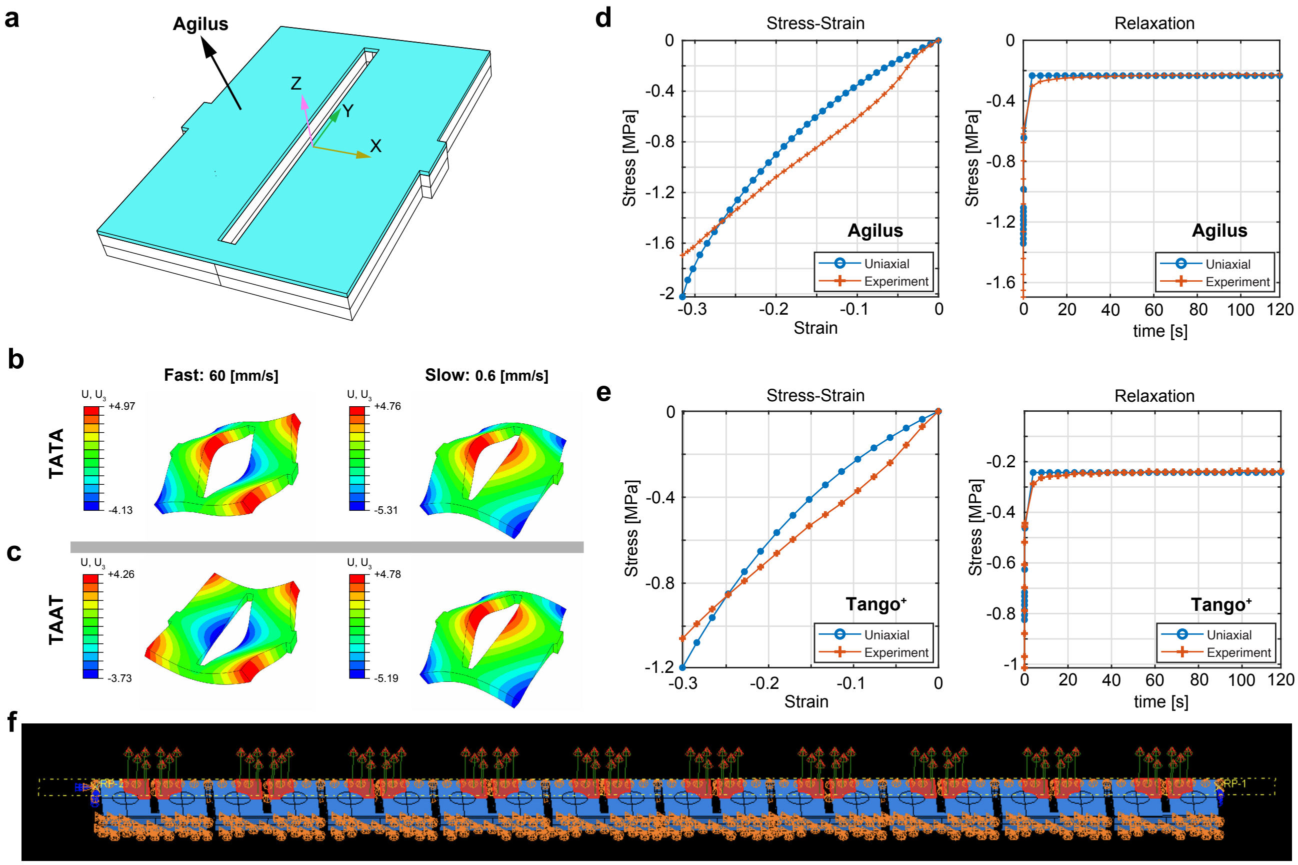

FIG. S2: **Computational prediction of strain rate dependent post-buckling of TATA and TAAT unit cells.** (a) In our computational analysis we used a standard-size unit cell covered with a finishing Agilus layer. (b, c) Our computational results confirm the manufacturability of TATA and TAAT designs using polyjet printing. (d, e) The fitting of the instantaneous and relaxation data to a single-term Prony series. (f) The loading condition used to mimic the geometrical imperfection observed in 3D printed strips.

D. Viscoelastic snap-back leading to traveling waves

Viscoelastic kirigami exhibit stress relaxation and for some regimes of strain, a viscoelastic snap-back (Fig. S4a and Video 2) is anticipated [3, 7, 8]. We quantify such snapping by tracking the relaxation of a TATA unit cell (300% of standard size unit cells, stretched 9 mm at 50 mm/s) using an optical tracking system called AruCo [9, 10]. In our experiments, four rigid tiles are embedded symmetrically within the geometry of the unit cell and marked with so called AruCo markers, which are small square monochrome images with specific patterns (Fig. S4b). The patterns are then used to identify the object and determine both the position and rotation of the markers. We track each tile separately by choosing unique markers on each tile.

While initially, the kirigami unit cell is deformed into the high-speed anti-symmetric mode, at longer times, it gradually creeps back into a symmetric mode (Fig. S4a and Video 2). Such transformation starts with a slow creep process, but over the course of 100 sec, the bottom half of the unit cell snaps back, such that the unit cell ultimately relaxes into the low-speed symmetric mode (Fig. S4c, blue curve). Such transformation can be manifested by switching the sign of the angle between the normal vectors at the middle of corresponding AruCo markers on the snappy half unit cell. In a limited range, the snap-back of viscoelastic strips is highly sensitive to geometrical imperfections. A slight change in the angle of rigid end-connections of the unit cell result in, for example, a shorter delay prior to a viscoelastic snap-back while there is not a visible change in the final angle ψ between the panels (Fig. S4c, green curve). That shows the strong influence of geometrical imperfections [2, 7] on the viscoelastic snap-trough of our kirigami.

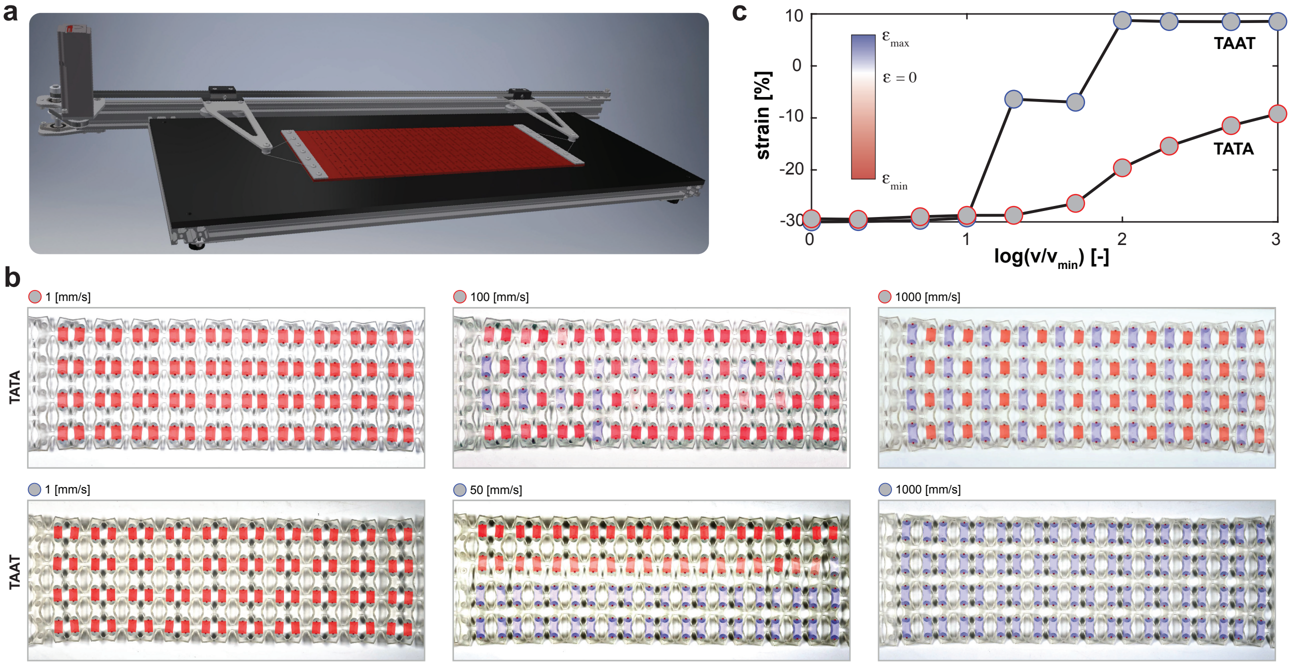

FIG. S3: **Strain rate sensitivity.** (a) The 3D configuration of fast-stretch setup equipped with a Teflon substrate. (b) The buckled geometry of TATA and TAAT kirigami stretched at different speeds. At intermediate range of speeds combined modes such as bidomain mode is observed. (c) Strain rate sensitivity of TAAT and TATA plates can be manifested according to the intermediate values of lateral strain between the maximum and minimum values of lateral strain.

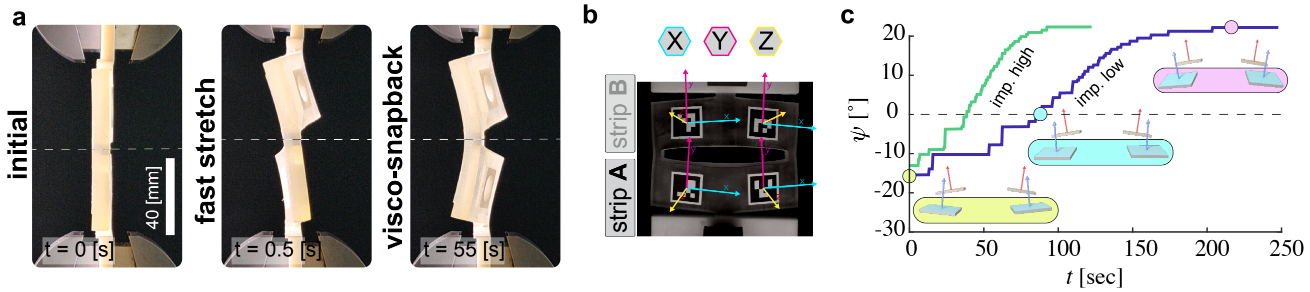

FIG. S4: **Viscoelastic snap-back.** (a) A TATA unit cell (300% of standard size, stretched 12.5 mm at 500 mm/s) exhibits a viscoelastic snap-back. (b) We used a tracking system to quantify the viscoelastic behavior of the TATA unit cell. (c) The overdamped snap-back of Strip'A' of a TATA unit cell (300% of standard size, stretched 9 mm at 50 mm/s) is quantified by the change of the angle between the normal vectors on the corresponding markers from positive to negative.

E. Quantifying the wave propagation in kirigami strips

To quantify the travelling of overdamped waves in our experiments, we measured the lateral straining of the snappy half unit cells, from their high-speed stretched configuration, by tracking two markers on their top sides using a custom Matlab code. While lateral strains exceed more than 6%, the snap-back of viscoelastic strips can be manifested based on a rapid change in the value of the lateral strain over time—as it is distinguishable according to the color codes in Fig. S5. The sequence of snap-backs reveals the existence of a mechanical kink wave travelling at a constant speed (Fig. S5b). Moreover, the time delay prior to the first snap-back (i.e., corresponding to strip no. 2) can be attributed to the size of imperfections at the corresponding boundary.

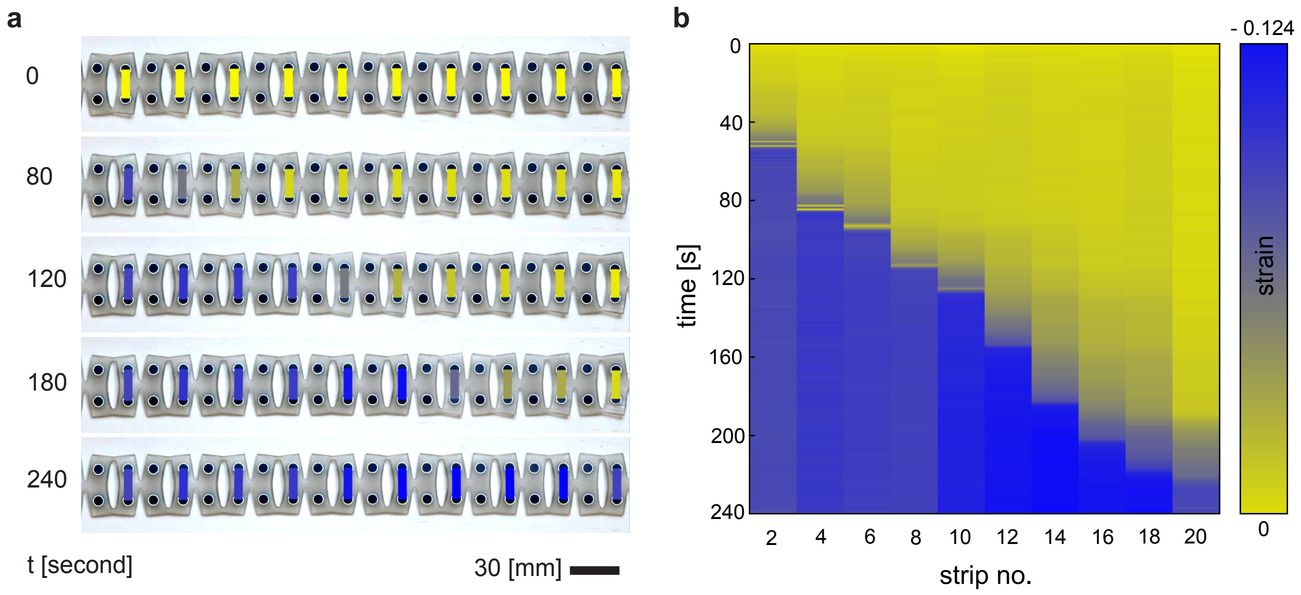

FIG. S5: **Non-linear travelling kink in a TATA kirigami strip.** (a) The process of snap-back in a kirigami strip. Yellow is corresponding to the high-speed (anti-symmetric) configuration of TATA unit cells and blue represents their low-speed (symmetric) mode. (b) Transition from high-speed mode to low-speed mode.

F. Dynamic shape-morphing kirigami

As an example of functional materials that exhibit dynamic shape-transformation, we showcase a kirigami, made by texturing TAAT unit cells, that transforms its texture over time (Fig. S6). While computationally we have the freedom to investigate a larger space of material properties, practically, we are limited to available photopolymers (i.e., Agilus and Tango^{+}) to fabricate such a kirigami. We, therefore, modulate the strain-rate dependency and the snapping properties of TAAT unit cells by slightly varying their geometry. We found that additional longitudinal short cut-lines (Fig. S6) minimizes the stiff interaction of the transversely arranged unit cells. Therefore, the unit cells have more freedom to snap-back while they initially show a high amplitude of out-of-plane buckling—while stretched at high speeds. We, then see that our design (made of 80% size unit cells) exhibits a dynamic shape-transformation, from a plus- to a minus pattern (Fig. 4b, Video 8) while it is initially stretch at a high speed (1000 mm/s).

G. Mimicking the behavior of Mimosa pudica

To mimic the touch-sensitive sequential folding of Mimosa Pudica leaves, we equipped a strip made of 12 identical TATA unit cells (made of standard-size unit cells flipped such that the Agilus finishing layer is at the bottom side) with 3D printed spile connectors that allow taping paper leaflets at the two sides of the snappy half unit cells (Fig. S7). The leaflets are light enough so that the kirigami can carry their weight. The two end unit cells do not have leaflets; instead, we glue two foam wedges objects to the snappy half unit cells to prevent the spontaneous wave initiation due to the boundary effects. We then see that TATA unit cells buckle into their anti-symmetric high-speed mode when the strip is stretched at a high speed—that mimics the open configuration of a Mimosa Pudica leaf. Upon touch, the first carrying half unit cell buckles downward, and the following wave emerges mimicking the sequential folding of Mimosa Pudica leaflets (Fig. 4a, Video 7).

H. Mass transportation

To demonstrate that travelling kinks can move objects, we equipped the 8 middle unit cells of a TATA strip made of 10 unit cells (130% of standard unit cells) arms and guiders made of rigid plastic (PLA) (Fig. S8). In addition, to make sure that the kirigami is able to carry the weight of the plastic arms and of the ping-pong ball while keeping a high enough amplitude of snap-back, we glued a hyperelastic strip on top of the Tango^{+} layer. The hyperelastic strips

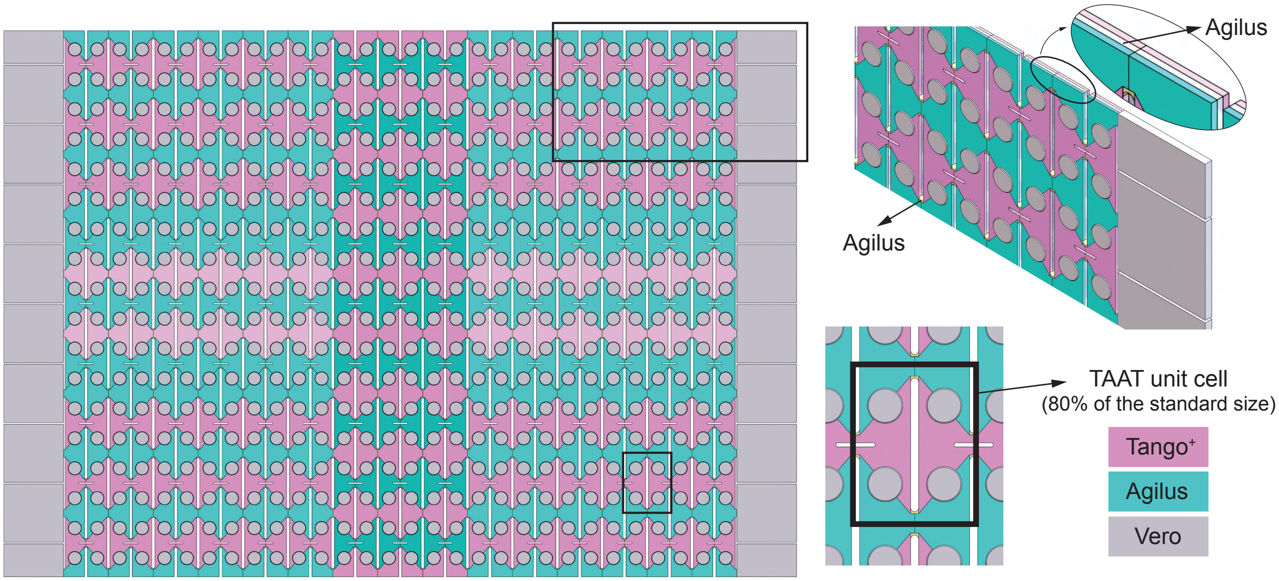

FIG. S6: **Multi-texture design of the plus-minus shape-morphable plate.** The longitudinal cut-lines minimize the stiff interaction of the transversely arranged unit cells that facilitate the snap-back of TAAT Unit cells.

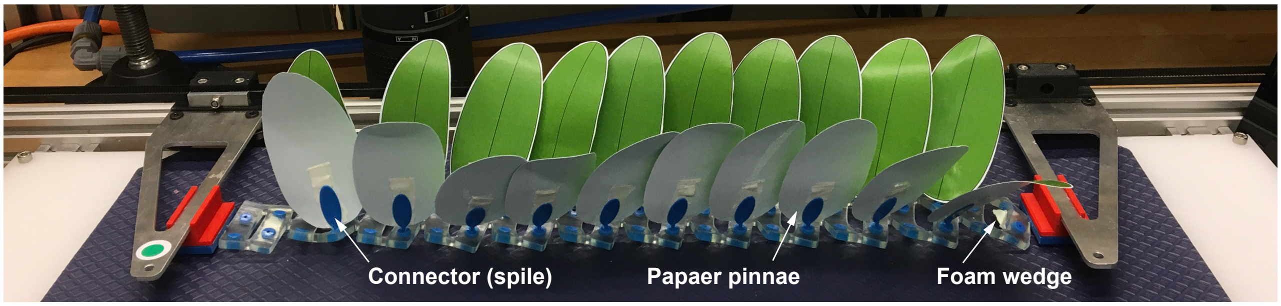

FIG. S7: **Setup to mimic the behavior of Mimosa pudica.** The assembly of a TATA kirigami strip carrying paper leaflets on our custom-made test setup.

are mold-cast using an addition silicone rubber (Elite Double 8 Normal, Zhermack) and have been glued to the TATA unit cells using silicone glue (Sil-Poxy, Smooth-On). Now, if we start from a high-speed mode (i.e., anti-symmetric mode), a sequential snapping is able to move a ping-pong ball using PLA arms and guiders(Fig. 5, Video 9).

I. Grasp and release

As an example of grasp and sequential release of objects, we produced a shorter TATA strip with three unit cells that carry stiff arms on their snapping half unit cells and two-pole kirigami bridges on their non-snapping half unit cells. In order to extend the amplitude of the snap-back, we glued a patch of silicone rubber (Dragon Skin™ 20, Smooth-On) on top of the Tango$^+$ layer of the snapping half unit cells (Fig. S9). The width of the silicone patches varies to ensure an effective sequential release independent of imperfections. Upon a fast stretch, the kirigami bridge and stiff arms deflect towards each other that make the grasping of ping-pong balls feasible (Video 10). Over time, the snapping half unit cells sequentially snap-back, resulting in a sequential release of ping-pong balls in accordance with a diffusive kink.

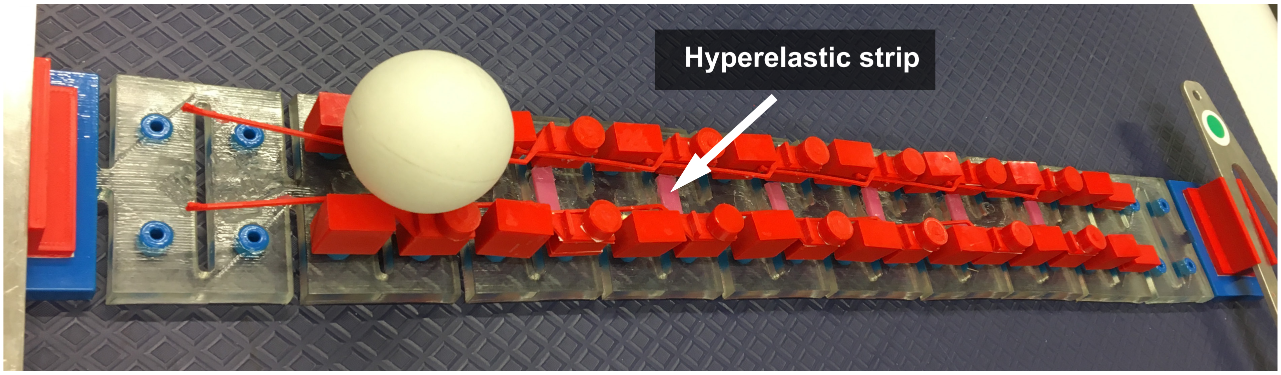

FIG. S8: **Setup to move a ping-pong ball.** The arms are mounted on the snappy half unit cells which their snap-back amplitude is enhanced using a hyperelastic strip. The not-snappy half unit cells carry the guiders.

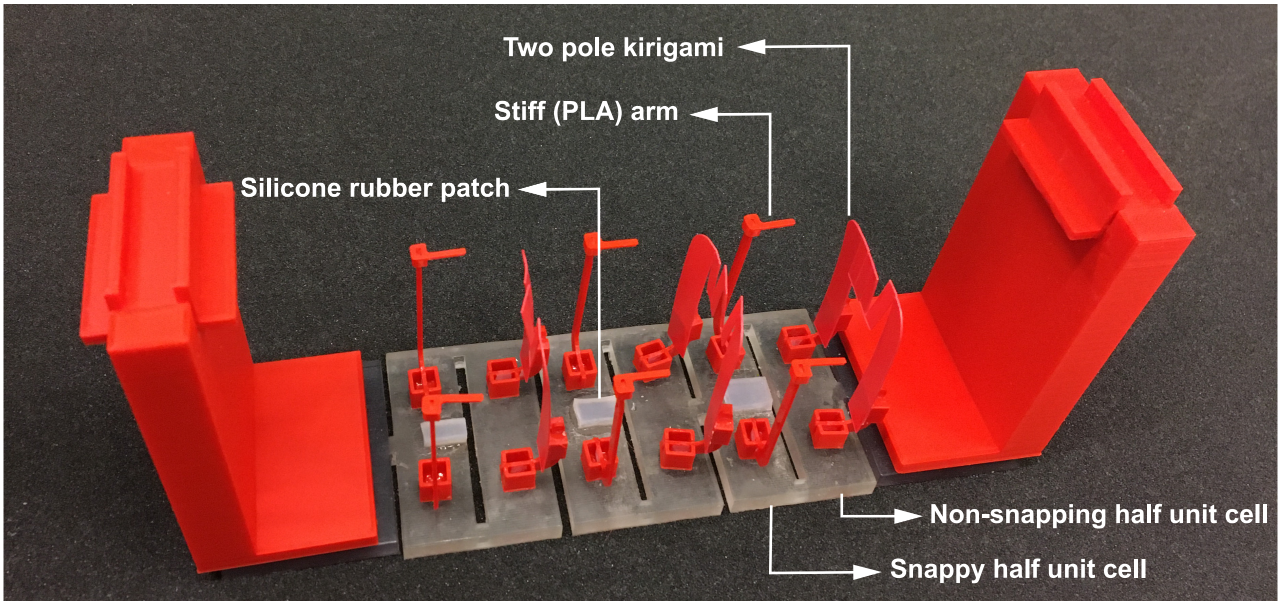

FIG. S9: **Grasp and release.** The stiff arms are mounted on the snapping half unit cells which their snap-back amplitude is enhanced using a hyperelastic strip. The non-snapping half unit cells carry the two-pole kirigami bridges.

J. Carrying spatial mechanisms

We integrated spatial four-bar linkages on the unit cells of a kirigami strip with defined elastic and viscoelastic properties (Fig. S10a). Following a defined loading procedure (a fast stretch followed by a low-speed stretch which is coincident with relaxation), our computational model simulates a rowing machine—as an example of soft robotic machines—that exhibit a diffusive kink after a fast stretch. Subsequently, a relatively slow unloading step completes the cycle (Video 11). Following the points P_i on the pedals carried by identical four-bar linkage mechanisms (Fig. S10b), we track their paths of motion. The effective cyclic motion of each point is influenced by the relative motions of the corresponding unit cell that can potentially be useful to perform differential functions.

K. Viscoelastic von Mises trusses

We use a viscoelastic von Mises truss to model the shape-transformation of individual viscoelastic kirigami unit cells (e.g., from a meta-stable anti-symmetric to a symmetric mode), and wave propagation in kirigami strips.

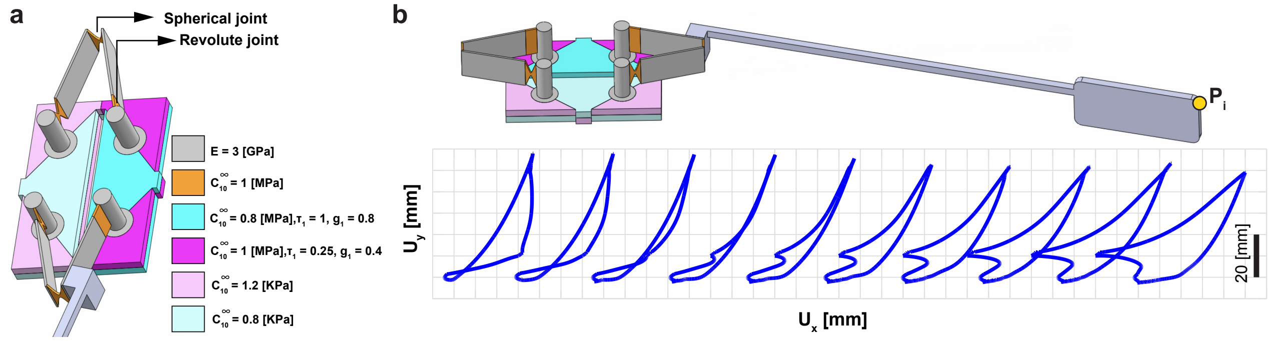

FIG. S10: **Spatial diffusive machines.** (a) The multimaterial design of the kirigami unit cell that is used in our rowing machine. (b) The computational modeling of a kirigami strip carrying pedals using integrated spatial four-bar linkages shows an example of soft robotic devices that moves its component on hysteretic paths according to a diffusive kink.

1. **Viscoelastic snap-back–0D Model**

The shape-transformation in a kirigami unit cell can be defined by the time-dependent snapping of one of its half-unit cells. Such a change of mode is similar to the pseudo-bistability of a popper geometry [7]. Excluding the effect of inertial forces, such time-dependent snapping can be modelled as the snapping of a simple van Mises truss made by joining a pair of linear springs $K/2$. A pre-stretched spring k and a dashpot c with a parallel configuration then connect the moving joint to the ground and transforms the system into a viscoelastic bi-stable configuration (Fig. S11). In such a model, the non-linearity is purely geometrical as it comes from the von Mises truss.

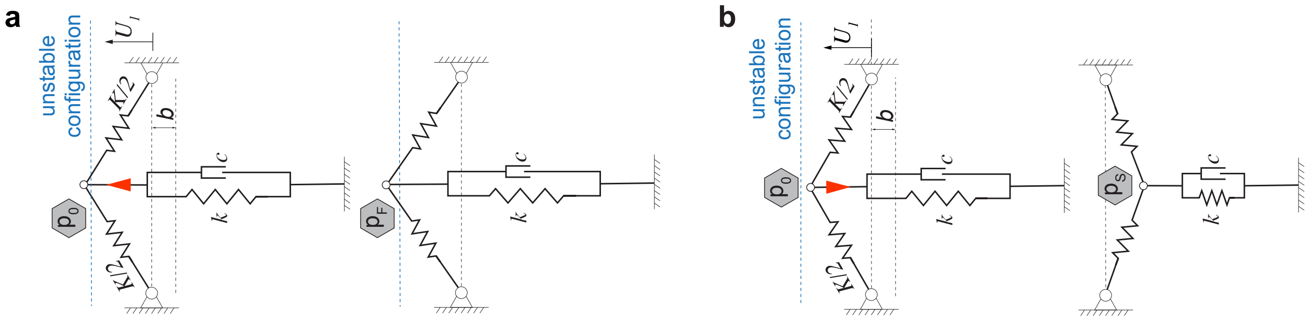

FIG. S11: **Bi-stable viscoelastic trusses.** The initial condition of a viscoelastic truss determines the (a) movement of the joint in a viscoelastic von Mieses truss towards the high-speed mode or results in a (b) snap-back.

To evaluate the dynamics of snapping we used a simplified Lagrangian formulation that comprises the elastic potential energy V and the Rayleigh dissipation function D:

$$-\frac{\partial V}{\partial U_i} + \frac{\partial D}{\partial \dot{U}_i} = 0, \tag{A1}$$

where U_i denotes the displacement of point P in the generalized coordinate i (i=1). Let's assume that due to an initial condition the joint P initially moves towards a point (P_0) close to the unstable configuration. Then, dependent on the offset from the unstable configuration our truss model may exhibit either a movement towards the high-speed stable configuration P_F (Fig. S11a) or a snap-back motion towards the low-speed stable configuration P_S (Fig. S11b). The potential energy of the truss can be, then, written as:

$$V(\vec{U}) = \frac{1}{2}k(U_1 + b)^2 + \frac{1}{2}K(\sqrt{a^2 + U_1{}^2} - d_0)^2, \tag{A2}$$

and the Rayleigh dissipation function formulated as:

$$D(\dot{\vec{U}}) = \frac{1}{2}c\dot{U}_1{}^2 \tag{A3}$$

where d_0 is the natural length of the springs $K/2$, a is half of the distance between the fixed joints and b is the offset from and straight configuration that describe the free stress configuration of spring k. By substituting the energy terms Eq. (A2) and Eq. (A3) in Eq. (A1), the equations of motion for the viscoelastic snapping can be driven as:

$$c\dot{U_1} = -KU_1 + K(\frac{d_0}{\sqrt{a^2 + {U_1}^2}})U_1 - k(U_1 + b) \tag{A4}$$

Then, to simplify the equation of motion, we use the leading terms of Taylor expansion of nonlinear terms centered at $X_i = 0$ that removes U_1 from the denominator in Eq. (A4) up to cubic order 3:

$$\dot{U_1} = (-\frac{K}{c} - \frac{k}{c} + \frac{Kd_0}{ca})U_1 - \frac{Kd_0}{2ca^3}U_1^3 - \frac{kb}{c} + \mathcal{O}({U_1}^4) \tag{A5}$$

To investigate the mechanics of viscoelastic snap-back regardless of geometry and material we nondimensinalize the equation of motion. Considering a characteristic time scale T:

$$\bar{t} = \frac{t}{T} \quad \longrightarrow \quad \frac{dU_1}{dt} = \frac{d\bar{t}}{dt}\frac{dU_1}{d\bar{t}} = \frac{1}{T}\frac{dU_1}{d\bar{t}},$$

therefore, the equation (A5) can be rewritten as:

$$U_{1,\bar{t}} = (-\frac{K}{c} - \frac{k}{c} + \frac{Kd_0}{ca})TU_1 - \frac{Kd_0T}{2ca^3}U_1^3 - \frac{kbT}{c}. \tag{A6}$$

the characteristic time scale time$T$ can be then chosen such that:

$$(-\frac{K}{c} - \frac{k}{c} + \frac{Kd_0}{ca})T = 1 \quad \longrightarrow \quad T = \frac{ca}{(-K-k)a + Kd_0},$$

therefore, (A6) can be rewritten as:

$$U_{1,\bar{t}} = U_1 - \frac{Kd_0}{2a^2((-K-k)a + Kd_0)}U_1^3 - \frac{kab}{((-K-k)a + Kd_0)}. \tag{A7}$$

We define the dimensionless coordinate $\bar{U_i}$ such that:

$$U_1 = \frac{kab}{((-K-k)a + Kd_0)}\bar{U_1}.$$

The dimensionless governing equation of motion can be written as:

$$\bar{U}_{1,\bar{t}} = \bar{U_1} - \beta\bar{U_1}^3 - 1 + \mathcal{O}(\bar{U_1}^4), \tag{A8}$$

where the dimensionless parameter $\beta = \frac{Kk^2b^2d_0}{2((-K-k)a+Kd_0)^3}$ is a function of material properties and nonlinear geometry.

2. Travelling kink–1D Model

In the next step, we investigate the mechanics of traveling waves in a kirigami strip which is made by connecting identical 0D trusses using linear elastic springs R assuring the compliant interaction of moving joints P_is (Fig. S12). The equation of motion for the ith joint can be derived in the same way as above, where:

$$\begin{aligned} V(\vec{U}) =& \frac{1}{2}k\sum_{i=1}^{N}(U_i + b)^2 + \frac{1}{2}K\sum_{i=1}^{N}(\sqrt{a^2 + {U_i}^2} - d_0)^2 + \frac{1}{2}R\sum_{i=1}^{N-1}(U_{i+1} - U_i)^2 \\ &+ \frac{1}{2}R\sum_{i=1}^{N-1}(U_i - U_{i-1})^2 \end{aligned} \tag{A9}$$

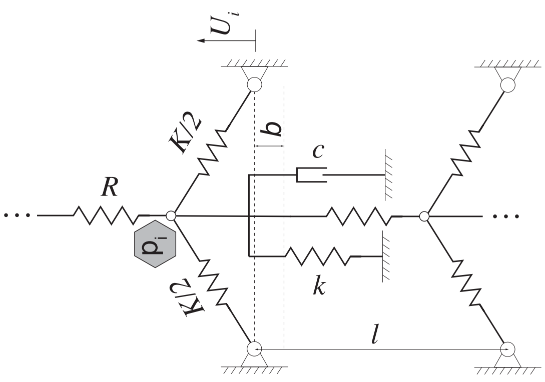

FIG. S12: **1D model.** Our 1D model is based on a series of viscoelastic von Mises trusses integrated into a string using the linear springs R.

and the Rayleigh dissipation function is:

$$D(\dot{\vec{U}}) = \frac{1}{2} c \sum_{i=1}^{N} (\dot{U}_i)^2 \tag{A10}$$

The equations of motion for the ith unit cell can be then written as:

$$c\dot{U}_i = -KU_i + K\left(\frac{d_0}{\sqrt{a^2 + {U_i}^2}}\right)U_i - k(U_i + b) + R(U_{i+1} - 2U_i + U_{i-1}) \tag{A11}$$

Furthermore, in order to simplify the equations of motion, we use the first two terms of Taylor expansion centered at $X_i = 0$ to remove the X_i from the denominator in Eq. (A11):

$$\dot{U}_i = \left(-\frac{K}{c} - \frac{k}{c} + \frac{Kd_0}{ca}\right)U_i - \frac{Kd_0}{2ca^3}U_i^3 - \frac{kb}{c} + \frac{R}{c}(U_{i+1} - 2U_i + U_{i-1}) + \mathcal{O}({U_i}^4) \tag{A12}$$

We then nondimensionalise the equation of motion, whereas by defining $U_i = \frac{kab}{((-K-k)a+Kd_0)}\bar{U}_i$ the governing equation of motion (14) can be written as:

$$\bar{U}_{i,\bar{t}} = \bar{U}_i - \beta\bar{U}_i^3 - 1 + L^2(\bar{U}_{i+1} - 2\bar{U}_i + \bar{U}_{i-1}) + \mathcal{O}(\bar{U}_i^4). \tag{A13}$$

in which $L^2 = \frac{Ra}{(-K-k)a+Kd_0}$. In the continuum limit, the generalized displacements $\bar{U}_{i+1}$ and $\bar{U}_{i-1}$ can be rewritten using a Taylor expansion as: $\bar{U}_i = \bar{U}(x)$ and $\bar{U}_{i\pm1} = \bar{U}(x) \pm \frac{\partial \bar{U}}{\partial \bar{x}} + \frac{1}{2}\frac{\partial^2 \bar{U}}{\partial \bar{x}^2} \pm \frac{1}{6}\frac{\partial^3 \bar{U}}{\partial \bar{x}^3} + \cdots$, Therefore, the dimensionless continuum limit of the equation of motion Eq. (A11) can be written as:

$$\bar{U}_{,\bar{t}} = \bar{U} - \beta\bar{U}^3 - 1 + \frac{\partial^2 \bar{U}}{\partial \bar{X}^2} + \mathcal{O}(U^4) \tag{A14}$$

when, $\bar{X} = L\bar{x}$ is the dimensionless coordinate.

[1] Bossart, A., Dykstra, D. M., van der Laan, J. & Coulais, C. Oligomodal metamaterials with multifunctional mechanics. *Proceedings of the National Academy of Sciences* **118** (2021).

[2] Janbaz, S., Narooei, K., Van Manen, T. & Zadpoor, A. Strain rate–dependent mechanical metamaterials. *Science Advances* **6**, eaba0616 (2020).

[3] Dykstra, D. M., Janbaz, S. & Coulais, C. The extreme mechanics of viscoelastic metamaterials. *APL Materials* **10**, 080702 (2022).

[4] Janbaz, S. *et al.* 3d printable strain rate-dependent machine-matter (2022). URL https://arxiv.org/abs/

2206.15168.

[5] Zhao, Z. *et al.* Origami by frontal photopolymerization. *Science Advances* **3**, e1602326 (2017).

[6] Eghbali, R. & Narooei, K. A hyperelastic-damage model to study the anisotropic mechanical behavior of coral-hydrogel bio-composite. *Journal of the Mechanical Behavior of Biomedical Materials* **126**, 105054 (2022).

[7] Gomez, M., Moulton, D. E. & Vella, D. Dynamics of viscoelastic snap-through. *Journal of the Mechanics and Physics of Solids* **124**, 781–813 (2019).

[8] Santer, M. Self-actuated snap back of viscoelastic pulsing structures. *International Journal of Solids and Structures* **47**, 3263–3271 (2010).

[9] Romero-Ramirez, F. J., Muñoz-Salinas, R. & Medina-Carnicer, R. Speeded up detection of squared fiducial markers. *Image and vision Computing* **76**, 38–47 (2018).

[10] Garrido-Jurado, S., Muñoz-Salinas, R., Madrid-Cuevas, F. J. & Medina-Carnicer, R. Generation of fiducial marker dictionaries using mixed integer linear programming. *Pattern Recognition* **51**, 481–491 (2016).
