# Supplementary material for: Diffusive kinks turn kirigami into machines
Source: Nat Commun. 2024 Feb 10;15:1255. doi: 10.1038/s41467-024-45602-7 (PMC10858914; doi:10.1038/s41467-024-45602-7)
Supplement: Supplementary file 3 — Description of Additional Supplementary Files [file 41467_2024_45602_MOESM3_ESM.pdf]

Description of Additional Supplementary Files

File Name: Supplementary Video 1
Description: Texture mediated by strain-rate

File Name: Supplementary Video 2
Description: Viscoelastic snap-back

File Name: Supplementary Video 3
Description: Propagation of a diffusive kink: simulation from Eq (2).

File Name: Supplementary Video 4
Description: Diffusive kink in a kirigami strip - FEM

File Name: Supplementary Video 5
Description: Diffusive kink in a kirigami strip - experiments

File Name: Supplementary Video 6
Description: Diffusive kink in a kirigami strip - experiments

File Name: Supplementary Video 7
Description: Mimicking Mimosa Pudica

File Name: Supplementary Video 8
Description: Dynamic morphing of a kirigami sheet

File Name: Supplementary Video 9
Description: Transporting an object with a diffusive kink

File Name: Supplementary Video 10
Description: Sequential release of multiple objects

File Name: Supplementary Video 11
Description: sequential actuation by a diffusive kink
